# Tobacco use and incident sleep parameters among a rural ageing population in South Africa

**DOI:** 10.18332/tid/156844

**Published:** 2023-01-20

**Authors:** Supa Pengpid, Karl Peltzer

**Affiliations:** 1 Department of Health Education and Behavioral Sciences, Faculty of Public Health, Mahidol University, Bangkok, Thailand; 2 Department of Public Health, Sefako Makgatho Health Sciences University, Pretoria, South Africa; 3 Department of Healthcare Administration, College of Medical and Health Science, Asia University, Taichung, Taiwan; 4 Department of Psychology, University of the Free State, Bloemfontein, South Africa; 5 Department of Psychology, College of Medical and Health Science, Asia University, Taichung, Taiwan

**Keywords:** tobacco use, sleep parameters, longitudinal study, South Africa

## Abstract

**INTRODUCTION:**

Tobacco use may be associated with incident insomnia. The aim of the study was to investigate the association between tobacco use and incident sleep parameters in a longitudinal study in South Africa.

**METHODS:**

Longitudinal data from two consecutive waves of middle-aged and older adults in 2014–2015 (n=5059) and 2018–2019 (n=4176) in rural South Africa were analyzed. Tobacco use and sleep parameters were assessed by self-report. The associations between tobacco use and incident sleep parameters were estimated with multivariable logistic regression.

**RESULTS:**

The prevalence of baseline sleep parameters was poor sleep quality 6.5%, sleep disturbance 13.6%, restless sleep 32.9%, and breathing stops 7.0%. In the fully adjusted model for people without poor sleep quality at baseline, daily tobacco smoking, smoking ≥10 units of tobacco products, current tobacco use and current smokeless tobacco use did not increase the odds of incident poor sleep quality. Smoking ≥10 units of tobacco products in a day (AOR=3.83; 95% CI: 1.77–8.28), current tobacco use (AOR=1.65; 95% CI: 1.09–2.51), and daily tobacco smoking (AOR=2.16; 95% CI: 1.15–4.07), were significantly positively associated with incident sleep disturbance. Furthermore, incident restless sleep was significantly positively associated with smoking ≥10 units of tobacco products in a day (AOR=3.97; 95% CI: 1.18–13.37), current smokeless tobacco use (AOR=2.78; 95% CI: 1.17–6.62) and current tobacco use (AOR=2.00; 95% CI: 1.00–4.00). Incident breathing stops were significantly positively associated with daily smoking tobacco (AOR=2.08; 95% CI: 1.11–3.34), smoking 1–9 units of tobacco products in a day (AOR=2.17; 95% CI: 1.20–3.94), and current tobacco use (AOR=1.77; 95% CI: 1.16–2.72).

**CONCLUSIONS:**

Higher tobacco use was independently associated with incident sleep disturbance, incident restless sleep, and incident breathing stops, but not with incident poor sleep quality.

## INTRODUCTION

Sub-optimal sleep constitutes a major public health burden globally such that one in four adults is dissatisfied with his/her sleep and almost one in ten has an insomnia disorder^1^. For example, in ageing adults in South Africa, 9.1% reported nocturnal sleep problems^2^, and among ageing rural South Africans 31.3% of men and 27.2% of women reported nocturnal sleep problems^3^. It would be important to identify modifiable behaviors, such as tobacco use, that can be altered to improve sleep quality and reduce their public health impact^4^.

In a recent systematic review of six cohort studies, regular smoking but not occasional smoking and past smoking significantly increased the odds of incident insomnia^5^. In an earlier systematic review of ten prospective studies smoking was associated with the risk of incident sleep-related issues (including insomnia, sleep disturbances, sleep apnea, sleep difficulty, narcolepsy, daytime sleepiness, and sleep complaints)^6^. However, all the studies in these two reviews^5,6^ had been conducted in high-income countries, and there is a lack of knowledge about the relationship between tobacco use and incident sleep disturbance in low- and middle-income countries including those in Africa. Cross-sectional evidence from central China showed that smokers had a higher prevalence of sleep disturbances than non-smokers^7^, while a cross-sectional study in the adult population of Indonesia showed a significant positive association between smoking and sleep disturbance^8^. In a cross-sectional study among university students in 27 countries, including African countries, less than daily and daily tobacco use increased the chances of restless sleep and poor sleep quality^9^. However, no significant association between tobacco use and sleep problems was found in adjusted analysis in a cross-sectional national adult population in South Africa^10^.

Some research has conceptualized how smoking affects sleep, for example, nicotine impacts on the release of neurotransmitters that affect the sleep-wake cycle^11^, nicotine withdrawal, which occurs while sleeping^12^. Medical consequences linked with smoking, such as chronic obstructive lung disease, can also impact on sleep negatively^13^. The aim of this study was to evaluate the association between tobacco use and incident sleep parameters in a longitudinal study in rural South Africa.

## METHODS

### Sample and procedures

Data were analyzed from two waves (2014–2015 and 2018–2019) of the ‘Health and Ageing in Africa: A Longitudinal Study of an INDEPTH Community in South Africa (HAALSI)’ in Agincourt. The number of participants in 2014–2015 was 5059 (aged ≥40 years) with a response rate of 85.9%^14^, and in 2018–2019, 4176 individuals participated in the 1st wave of the survey with a 94% response rate [12% (595) had died at follow-up, 5% (254) declined to participate, and <1% (34) could not be traced]. Further study procedures are shown elsewhere^14^. Participants were interviewed at home by trained field workers using computer-assisted personal interviewing (CAPI)^14^. The study protocol was approved by the University of the Witwatersrand Human Research Ethics Committee, the Harvard T.H. Chan School of Public Health, Office of Human Research Administration, and the Mpumalanga Provincial Research and Ethics Committee^14^. Participants provided written informed consent.

### Measures

*Outcome variables*

Poor sleep quality was measured with one item: ‘Over the past 4 weeks, how would you rate your sleep quality overall? Would you say it is very good, fairly good, fairly bad, or very bad?’. Poor sleep quality was classified as ‘fairly bad, or very bad’^15^. Sleep disturbance was sourced from the item: ‘During the past 4 weeks, how often did you wake up in the middle of the night or early morning?’. Response options were: 1=never, 2=less than once a week, 3=once or twice a week, and 4=three or more times a week’. Sleep disturbance was defined as awakening ≥3 times/week^15^.

Restless sleep was sourced from the item: ‘Much of the time in the past week, your sleep was restless’, ‘Yes’ in survey 1 and ‘most of the time or always’ in survey 2^16^.

Breathing stops were sourced from: ‘During the last month, have you had, or ever been told that your breathing stops or you struggle for breath?’ (Yes)^16^.

*Exposure variables*

Exposure to tobacco products was sourced from: ‘Have you ever smoked any tobacco product such as cigarettes, cigars or pipes?’ (Yes/No), and ‘Do you currently smoke any tobacco products, such as cigarettes, cigars, or pipes?’ (Yes/No). Current smokers were asked: ‘How often do you smoke tobacco products?’ with response options ‘daily, 5–6 days per week, 1–4 days per week, 1–3 days per month, less than once per month’, which were grouped into daily and non-daily; and ‘On days you smoke, how many of the following do you smoke per day?’ with choices including cigars, cheroots, hand-rolled cigarettes, manufactured cigarettes, pipes full of tobacco, and other. Responses were summed and grouped into: 0, 1–9 and ≥10 units of smoking tobacco per day. Participants were further asked: ‘At what age did you totally quit smoking or consuming tobacco’. Smokeless tobacco use was sourced from two items: ‘Have you ever used any smokeless tobacco such as snuff, chewing tobacco, snus, betel with tobacco?’ (Yes/No), and ‘Do you currently use smokeless tobacco products?’ (Yes/No)^14^.

*Covariates*

Sociodemographic data included education level, sex, age, migration, wealth and marital status of the household^14^.

Alcohol dependence was measured using the 4-item CAGE scale^17^ (Cronbach’s alpha 0.82).

Physical activity levels were classified according to Global Physical Activity Questionnaire (GPAQ) into low, moderate, and high (<600, 600–1500, and >1500 MET-minutes/week, respectively)^18^.

Sedentary behavior included the item ‘time usually spend sitting or reclining on a typical day?’ from the GPAQ^19^, and grouped into ‘<4 hours, 4 to <8 hours and ≥8 hours per day’^20^. Body mass index (BMI, kg/m^2^) was measured and classified according to WHO criteria^21^. Hypertension was measured and defined based on National Committee criteria^22^. Dyslipidemia was defined as: ‘total cholesterol >6.21 mmol/L, HDL-C <1.19 mmol/L, LDL-C >4.1 mmol/L, triglycerides >2.25 mmol/L, or ever diagnosed or medication use for high cholesterol’^14^. Diabetes was ‘classified with fasting glucose (defined as >8 hours) >7mmol/L (126 mg/dL), ever diagnosed or medication use for diabetes’^14^.

### Statistical analysis

Frequencies and percentages of people with incident sleep parameters were calculated. The first, second, third, and fourth logistic regression models excluded those with poor sleep quality, sleep disturbance, restless sleep, and breathing stops, respectively, at baseline, leaving an analytic sample of 3367, 3361, 2644 and 3620, respectively, to estimate incident poor sleep quality, incident sleep disturbance, incident restless sleep, and incident breathing stops, respectively. Tobacco use variables were the main predictors, and outcome variables included incident poor sleep quality, incident sleep disturbance, incident restless sleep, and incident breathing stops. Logistic regression models included four steps: unadjusted model, Model 1: adjusted for sociodemographic variables (education level, sex, age, migration, wealth and marital status); Model 2: adjusted for Model 1 variables plus behavioral variables (alcohol use, sedentary behavior, body mass index and physical activity); and Model 3: adjusted for model 1 and 2 variables, plus chronic conditions (diabetes, hypertension, and dyslipidemia). Longitudinal analyses applied inverse probability weights accounting for attrition and mortality at follow-up. A p<0.05 was considered significant. StataSE 15.0 (College Station, TX, USA) was used for the analyses.

## RESULTS

### Sample characteristics by incident sleep parameters

The prevalence of baseline sleep parameters was poor sleep quality 6.5%, sleep disturbance 13.6%, restless sleep 32.9%, and breathing stops 7.0%, while the prevalence of incident sleep parameters was poor sleep quality 13.9%, sleep disturbance 6.0%, restless sleep 2.7%, and breathing stops 4.8% (Table 1).

**Table 1:** Sample characteristics by incident sleep parameters, Agincourt, South Africa, 2014–2019 (N=5059)

*Baseline variables*		*Baseline sample*	*Incident poor sleep quality*	*Incident sleep disturbance*	*Incident restless sleep*	*Incident breathing stops*
**All**		N=5059	N=3677	N=3361	N=2644	N=3620
		***n (%)***	***%***	***%***	***%***	***%***
**Age** (years)	40–49	884 (17.6)	14.2	4.6	3.6	3.1
	50–59	1358 (27.1)	13.9	5.7	3.1	4.1
	60–69	1274 (25.4)	15.0	5.9	2.1	5.1
	70–79	918 (18.3)	13.1	7.8	1.9	5.7
	≥80	583 (11.6)	12.4	5.4	3.5	6.9
**Sex**	Female	2713 (53.6)	13.9	5.9	3.1	4.8
	Male	2346 (46.4)	13.9	6.1	2.4	4.7
**Country of birth**	Mozambique/other	1519 (30.2)	13.1	5.1	2.9	5.1
	South Africa	3508 (69.8)	14.2	6.1	2.7	4.6
**Education level** (years)	None	2307 (49.1)	13.5	6.0	2.7	5.4
	1–7	1613 (32.0)	13.6	5.7	2.6	5.1
	8–11	537 (10.7)	13.9	6.3	3.3	2.8
	≥12	585 (11.6)	15.8	6.1	2.6	3.3
**Marital status**	Married/cohabiting	2575 (50.9)	13.7	6.0	2.7	4.6
	Not married	2480 (49.1)	14.0	6.0	2.9	5.0
**Wealth index**	Low	2047 (40.5)	13.7	5.6	3.1	5.2
	Middle	991 (19.6)	15.2	7.8	3.6	3.9
	High	2021 (39.9)	13.5	5.4	2.0	4.7
**Alcohol dependence**	No	4988 (98.7)	13.9	6.0	2.7	4.8
	Yes	68 (1.3)	13.6	1.8	2.6	5.2
**Physical activity**	Low	221 (44.0)	13.1	6.2	3.2	5.5
	Moderate	1143 (22.7)	13.0	6.1	3.0	4.4
	High	1674 (33.3)	15.3	5.6	2.1	4.0
**Sedentary behavior**	Low	2675 (55.9)	13.8	6.2	2.3	4.3
	Moderate	1632 (34.1)	14.9	5.6	3.2	4.5
	High	475 (9.9)	12.6	5.6	3.9	7.1
**Body mass index**	Normal	1719 (36.7)	14.2	5.3	3.4	4.4
	Underweight	258 (5.5)	11.5	6.5	4.0	7.1
	Overweight	1328 (28.3)	12.9	6.1	2.9	4.5
	Obese	1384 (29.5)	15.2	6.0	1.6	4.7
**Hypertension**	No	2052 (41.6)	13.7	5.4	2.8	4.1
	Yes	2884 (58.4)	14.0	6.1	2.5	5.4
**Diabetes**	No	4093 (88.0)	13.6	5.9	2.7	5.0
	Yes	559 (12.0)	15.8	5.8	3.2	3.5
**Dyslipidaemia**	No	2389 (56.2)	13.1	6.2	2.4	4.7
	Yes	1862 (43.8)	14.8	5.9	3.2	5.1
**Smoking tobacco**	Never	3973 (78.7)	13.6	5.7	2.8	4.4
	Ever	431 (8.5)	15.2	5.5	2.2	4.0
	Quit	186 (3.7)	14.1	7.2	3.0	9.1
	Non-daily	91 (1.8)	15.9	8.6	2.0	7.0
	Daily	369 (7.3)	14.0	7.2	3.1	7.2
**Frequency of smoking tobacco products per day**	0	4596 (91.1)	13.9	5.8	2.7	4.5
	1–9	325 (6.4)	14.4	5.8	1.0	8.1
	≥10	124 (2.5)	13.9	14.0	7.8	5.2
**Smokeless tobacco use**	Never	4608 (91.2)	14.0	5.9	2.4	4.5
	Ever	108 (2.1)	11.5	7.1	3.8	11.1
	Current	339 (6.7)	12.4	5.5	7.1	6.7
**Current tobacco use**	No	4264 (84.4)	13.9	5.8	2.5	4.4
	Yes	790 (15.6)	13.3	6.7	4.5	7.0

### Correlations of tobacco use with incident poor sleep quality

In the fully adjusted model for people without poor sleep quality at baseline, daily tobacco smoking, smoking ≥10 units of tobacco products, current tobacco and current smokeless tobacco use, did not increase the odds of incident poor sleep quality (Table 2).

**Table 2:** Longitudinal association between tobacco use and incident poor sleep quality, Agincourt, South Africa, 2014–2019 (N=3677)

*Variable*	*Unadjusted model*	*Model 1*	*Model 2*	*Model 3*
	*OR (95% CI)*	*AOR (95% CI)*	*AOR (95% CI)*	*AOR (95% CI)*
**Smoking tobacco**				
Never (Ref.)		1	1	1
Ever	1.13 (0.84–1.53)	1.16 (0.86–1.57)	1.16 (0.85–1.58)	1.14 (0.82–1.58)
Quit	1.14 (0.71–1.82)	1.14 (0.71–1.84)	1.21 (0.74–1.98)	1.16 (0.67–2.01)
Non-daily	1.15 (0.62–2.11)	1.16 (0.63–2.13)	1.13 (0.61–2.09)	1.15 (0.58–2.23)
Daily	1.04 (0.74–1.44)	1.05 (0.75–1.46)	0.97 (68–1.38)	0.85 (0.56–1.28)
**Frequency of smoking tobacco products per day**				
0 (Ref.)		1	1	1
1–9	1.04 (0.74–1.47)	1.05 (0.74–1.48)	0.95 (0.66–1.37)	0.89 (0.59–1.36)
≥10	1.03 (0.59–1.81)	1.03 (0.59–1.82)	1.02 (0.58–1.81)	0.82 (0.42–1.64)
**Smokeless tobacco use**				
Never (Ref.)		1	1	1
Ever	0.82 (0.43–1.58)	0.84 (0.43–1.62)	0.76 (0.37–1.56)	0.70 (0.33–1.51)
Current	0.87 (0.60–1.27)	0.90 (0.62–1.33)	0.94 (0.64–1.40)	0.87 (0.55–1.35)
**Current tobacco use**				
No (Ref.)		1	1	1
Yes	0.95 (0.75–1.21)	0.97 (0.76–1.24)	0.95 (0.73–1.23)	0.85 (0.63–1.44)

AOR: adjusted odds ratio. Model 1: Adjusted for age, sex, education level, migration, marital and wealth status. Model 2: Adjusted for Model 1 variables, plus alcohol use, physical activity, sedentary behavior and body mass index. Model 3: Adjusted for Model 1 and 2 variables, plus dyslipidemia, hypertension, and diabetes. *p<0.05; **p<0.01; ***p<0.001.

### Associations between tobacco use and incident sleep disturbance

Table 3 shows, based on the longitudinal analysis, the associations between tobacco use and incident sleep disturbance. In fully adjusted models, smoking ≥10 units of tobacco products per day (AOR=3.83; 95% CI: 1.77–8.28), current tobacco use (AOR=1.65; 95% CI: 1.09–2.51), and daily tobacco smoking (AOR=2.16; 95% CI: 1.15–4.07), were significantly positively associated with incident sleep disturbance. Current smokeless tobacco use was not significantly associated with incident sleep disturbance (Table 3).

**Table 3:** Longitudinal association between tobacco use and incident sleep disturbance, Agincourt, South Africa, 2014–2019 (N=3361)

*Variable*	*Unadjusted model*	*Model 1*	*Model 2*	*Model 3*
	*OR (95% CI)*	*AOR (95% CI)*	*AOR (95% CI)*	*AOR (95% CI)*
**Smoking tobacco**				
Never (Ref.)		1	1	1
Ever	0.95 (0.59–1.53)	1.01 (0.60–1.69)	0.91 (0.50–1.65)	1.07 (0.58–1.98)
Quit	0.97 (0.34–2.74)	0.95 (0.33–2.76)	1.24 (0.42–3.65)	1.53 (0.51–4.59)
Non-daily	1.64 (0.76–3.55)	1.88 (0.84–4.23)	2.28 (0.98–5.29)	3.13 (1.30–7.58)*
Daily	1.30 (0.82–2.07)	1.48 (0.88–2.49)	2.06 (1.18–3.60)*	2.16 (1.15–4.07)*
**Frequency of smoking tobacco products per day**				
0 (Ref.)		1	1	1
1–9	0.97 (0.57–1.66)	1.07 (0.61–1.88)	1.42 (0.78–2.56)	1.71 (0.91–3.22)
≥10	2.70 (1.48–4.90)***	3.05 (1.62–5.76)***	4.15 (2.13–8.09)***	3.83 (1.77–8.28)***
**Smokeless tobacco use**				
Never (Ref.)		1	1	1
Ever	1.21 (0.52–2.81)	1.17 (0.50–2.74)	1.61 (0.68–3.84)	1.80 (0.75–4.34)
Current	0.95 (0.55–164)	0.93 (0.53–1.64)	1.16 (0.65–2.08)	1.13 (0.60–2.13)
**Current tobacco use**				
No (Ref.)		1	1	1
Yes	1.17 (0.83–1.65)	1.20 (0.84–1.73)	1.55 (1.06–2.28)*	1.65 (1.09–2.51)*

AOR: adjusted odds ratio. Model 1: Adjusted for age, sex, education level, migration, marital and wealth status. Model 2: Adjusted for Model 1 variables, plus alcohol use, physical activity, sedentary behavior and body mass index. Model 3: Adjusted for Model 1 and 2 variables, plus dyslipidemia, hypertension, and diabetes.

* p<0.05;

** p<0.01;

*** p<0.001.

### Associations between tobacco use and incident restless sleep

In fully adjusted models, smoking ≥10 units of tobacco products per day (AOR=3.97; 95% CI: 1.18–13.37), current smokeless tobacco use (AOR=2.78; 95% CI: 1.17–6.62), and current tobacco use (AOR=2.00; 95% CI: 1.00–4.00), were significantly positively associated with incident restless sleep. Daily smoking of tobacco was not significantly associated with incident restless sleep (Table 4).

**Table 4:** Longitudinal association between tobacco use and incident restless sleep, Agincourt, South Africa, 2014–2019 (N=2644)

*Variable*	*Unadjusted model*	*Model 1*	*Model 2*	*Model 3*
	*OR (95% CI)*	*AOR (95% CI)*	*AOR (95% CI)*	*AOR (95% CI)*
**Smoking tobacco**				
Never		1	1	1
Ever	0.80 (0.34–1.88)	1.10 (0.40–3.04)	0.84 (0.28–2.59)	0.79 (0.25–2.48)
Quit	0.92 (0.22–2.89)	1.20 (0.27–5.30)	0.66 (0.09–5.16)	0.63 (0.08–4.99)
Non-daily	0.87 (0.14–5.60)	1.09 (0.14–8.57)	1.08 (0.14–8.61)	1.34 (0.16–11.19)
Daily	1.20 (0.55–2.64)	1.35 (0.56–3.25)	1.33 (0.52–3.35)	1.14 (0.38–3.37)
**Frequency of smoking tobacco products per day**				
0		1	1	1
1–9	0.49 (0.13–1.86)	0.52 (0.13–2.06)	0.51 (0.12–2.13)	0.35 (0.05–2.57)
≥10	4.18 (1.67–10.44)**	4.50 (1.63–12.41)**	4.60 (1.55–13.65)**	3.97 (1.18–13.37)*
**Smokeless tobacco use**				
Never		1	1	1
Ever	1.64 (0.44–6.13)	2.13 (0.56–8.13)	2.73 (0.69–10.78)	2.89 (0.72–11.65)
Current	2.70 (1.43–5.09)**	3.60 (1.79–7.22)***	2.89 (1.33–6.27)**	2.78 (1.17–6.62)*
**Current tobacco use**				
No		1	1	1
Yes	1.91 (1.13–3.22)*	2.15 (1.23–3.77)**	1.88 (1.01–3.52)*	2.00 (1.00–4.00)*

AOR: adjusted odds ratio. Model 1: Adjusted for age, sex, education level, migration, marital and wealth status. Model 2: Adjusted for Model 1 variables, plus alcohol use, physical activity, sedentary behavior and body mass index. Model 3: Adjusted for Model 1 and 2 variables, plus dyslipidemia, hypertension, and diabetes.

* p<0.05;

** p<0.01;

*** p<0.001.

### Associations between tobacco use and incident breathing stops

In fully adjusted models, daily tobacco smoking (AOR=2.08; 95% CI: 1.11–3.34), smoking 1–9 units of tobacco products per day (AOR=2.17; 95% CI: 1.20–3.94), and current tobacco use (AOR=1.77; 95% CI: 1.16–2.72), were significantly positively associated with incident breathing stops. Current smokeless tobacco use was not significantly associated with incident breathing stops (Table 5).

**Table 5:** Longitudinal association between tobacco use and incident breathing stops, Agincourt, South Africa, 2014–2019 (N=3620)

*Variable*	*Unadjusted model*	*Model 1*	*Model 2*	*Model 3*
	*OR (95% CI)*	*AOR (95% CI)*	*AOR (95% CI)*	*AOR (95% CI)*
**Smoking tobacco**				
Never (Ref.)		1	1	1
Ever	0.89 (0.52–1.54)	0.85 (0.49–1.49)	0.83 (0.45–1.54)	0.51 (0.42–1.54)
Quit	1.02 (0.34–3.04)	1.03 (0.34–3.10)	0.88 (0.23–3.39)	0.97 (0.25–3.73)
Non-daily	1.61 (0.69–3.77)	1.70 (0.72–4.01)	1.99 (0.82–4.81)	1.88 (0.70–5.07)
Daily	1.68 (1.05–2.70)*	1.69 (1.03–2.76)*	2.06 (1.18–3.58)*	2.08 (1.11–3.34)*
**Frequency of smoking tobacco products a day**				
0 (Ref.)		1	1	1
1–9	1.85 (1.16–2.93)**	1.88 (1.16–3.03)**	2.21 (1.30–3.77)**	2.17 (1.20– 3.94)*
≥10	1.12 (0.45–2.83)	1.21 (0.48–3.08)	1.51 (0.58–3.94)	1.48 (0.40–4.37)
**Smokeless tobacco use**				
Never (Ref.)		1	1	1
Ever	2.64 (1.29–5.38)**	2.19 (1.06–4.05)*	1.99 (0.85–4.67)	2.21 (0.94–5.24)
Current	1.56 (0.95–2.57)	1.32 (0.79–2.22)	1.33 (0.75–2.36)	1.43 (0.77–2.64)
**Current tobacco use**				
No (Ref.)		1	1	1
Yes	1.67 (1.19–2.35)*	1.56 (1.10–2.23)*	1.77 (1.19–2.62)**	1.77 (1.16–2.72)**

AOR: adjusted odds ratio. Model 1: Adjusted for age, sex, education level, migration, marital and wealth status. Model 2: Adjusted for Model 1 variables, plus alcohol use, physical activity, sedentary behavior and body mass index. Model 3: Adjusted for Model 1 and 2 variables, plus dyslipidemia, hypertension, and diabetes.

* p<0.05;

** p<0.01;

*** p<0.001.

## DISCUSSION

In this novel longitudinal investigation in South Africa, we found that higher tobacco use was independently associated with incident sleep disturbance, incident restless sleep, and incident breathing stops, but not incident poor sleep quality. These findings seem to be largely consistent with a recent systematic review that, regular smoking but not occasional smoking and past smoking significantly increased the odds of incident insomnia^5^, and evidence from another systematic review and meta-analysis that smoking was associated with incident sleep-related issues^6^. The possible negative effect of tobacco use on sleep may be associated with the nicotine-enhancing effects on wake arousal^5,23,24^. ‘Cigarette smoking stimulates the release of dopamine (DA) and serotonin (5-HT), which might promote awakening and inhibit rapid eye movement sleep’^25^. While asleep, tobacco users may experience temporary nicotine withdrawal, leading to awaking mid-sleep^12^. Nicotine has also been identified as inhibiting sleep-promoting neurotransmitters^26^. Some evidence is also provided for a genetic correlation between smoking and sleep behavior, for example, a higher frequency of cigarette smoking affected the circadian rhythm by shifting from a more morning person to a more evening person, increasing insomnia^27^.

Consistent with a previous review^5^, we did not find an association between ex-smokers compared to never smokers and sleep parameters, suggesting that smoking cessation may be beneficial to sleep health. We found that daily smoking was associated with incident breathing stops, and heavy smoking was associated with incident restless sleep. Similarly, in a polysomnographical analysis, smokers developed more leg movements and sleep apneas in sleep than non-smokers^28^. In the unadjusted and first logistic regression model, ever smokeless tobacco use increased the odds of incident breathing stops. One explanation for this could be that participants with a history of snuff or smokeless tobacco use used the latter for medicinal reasons, including for pain conditions and insomnia^29^.

Our study found that current tobacco use (smoking and/or smokeless tobacco use) increased the odds of incident sleep disturbance, incident restless sleep and incident breathing stops. Similarly, in a large American cross-sectional adult survey, compared to non-tobacco users, those who were past month smokers and smokeless tobacco users had significantly higher odds of insufficient sleep^30^. Further, we found that current smokeless tobacco use was associated with incident restless sleep, while in a cross-sectional study among the general adult population in Sweden, smokeless tobacco (snus) use increased the odds of snoring^31^. More research is needed on the relationship between smokeless tobacco use and sleep disturbance.

Tobacco use is a major public health problem affecting the morbidity and mortality of large populations worldwide^32^. Consequently, understanding the different health consequences of tobacco use is central to public health^6^. This study showed that tobacco use is a risk factor for several sleep-related issues. Sleep disturbances constitute a major mental health burden^1^.

Tobacco users could be informed about a link between tobacco use and poor sleep parameters, and that tobacco use cessation could also be beneficial for sleep. Tobacco use cessation treatments, such as nicotine replacement therapies, may only be moderately effective, so sleep therapy may be a useful adjunct to smoking treatment^27,33,34^. In the management of sleep disorders, cessation of tobacco use could be offered as an adjunct.

### Limitations

Some data, including sleep parameters, were assessed by self-report and not verified by actigraphy or polysomnography, which may have led to an over- or under-estimation of sleep parameters. In a previous study, the same smokers demonstrated lower sleep quality measured objectively by actigraphy, but not according to the Pittsburg Sleep Quality Index (PSQI)^35^. Only single-item measures were used to assess sleep problems and future studies should use the DSM criteria. Tobacco use was assessed by self-report and future studies should measure nicotine levels and other chemicals in tobacco in people with sleep disturbances. Due to the small prevalence of tobacco use among women, we were unable to perform subgroup analyses by sex.

## CONCLUSIONS

Higher tobacco use was independently associated with incident sleep disturbance, incident restless sleep, and incident breathing stops, but not incident poor sleep quality. Tobacco use cessation management may want to include the treatment of sleep disturbance.

## Data Availability

The data supporting this research are available from the following sources: Harvard Center for Population and Development Studies (HCPDS) program website (www.haalsi.org).
